# Tissue reactivity and suture handling characteristics of “jimat” against silk and chromic gut in cat thigh muscle: A comparative study

**DOI:** 10.14202/vetworld.2015.958-969

**Published:** 2015-08-09

**Authors:** Tilahun Bekele, A. P. Bhokre, Abreha Tesfaye

**Affiliations:** 1 School of Veterinary Medicine, College of Agriculture and Veterinary Medicine, Jimma University, P. O. Box 307, Jimma, Ethiopia; 2 Department of Veterinary Medicine, College of Veterinary Medicine, Mekelle University, P. O. Box 231, Mekelle, Ethiopia

**Keywords:** handling characteristics, histopathology, leukocytic infiltration, sutures, thigh muscle

## Abstract

**Aim:**

This study was conducted to evaluate and compare the tissue reactivity and suture handling characteristics of chromic gut, silk, and ‘jimat’ suture materials in cat thigh muscle.

**Materials and Methods:**

This experimental study was conducted from November, 2013 to April, 2014 in Kombolcha Animal Diseases Survey, Research and Diagnostic Laboratory, Kombolcha, Ethiopia. A total of 36 local breed male cats were randomly assigned into chromic gut, silk, and “jimat” groups of 12 cats each as A, B, and C, respectively. The hind leg muscle biceps femoris was incised and sutured with suture materials according to their groups. The muscle samples with its suture were collected at six different days interval i.e. 1, 3, 7, 14, 21, and 28 and processed histopathologically to assess the degree of leukocytic infiltration and fibrous and granulation tissue formation (GTF). In addition, all suture materials were evaluated intraoperatively about their handling characteristics, by rating the precision of knot tying, square knot positioning, and resistance to knot slippage. The statistical analysis was done with two-way ANOVA, Kruskal–Wallis, and Chi-square tests.

**Results:**

The histopathology showed that “jimat” thread (2.4±1.2) had produced least leukocytic infiltration than chromic gut (4.5±1.9) and silk (4.3±1.5) sutures during the study period. Higher GTF was seen at day 3 (6 [100%]), 7 (6 [100%]) and day 14 (4 [66.7%]) in all sutures, whereas “jimat” showed significantly (p<0.05) higher fibrous tissue formation (10 [83.3%]) than others. Moreover, “jimat” suture had equal suture handling characteristics (p>0.05) with both chromic gut and silk.

**Conclusion:**

The result indicated that a single strand “jimat” thread appears to be the most satisfactory suture material as regards to both tissue reaction and suture handling characteristics for skeletal muscle approximation in cats and provided that studies on its carcinogenic effects should be done.

## Introduction

Use of suture materials for wound closure is an ancient art that dates back to Egyptian scrolls of 3500 BC that describe the use of linen to close wound edges [1,2]. Sutures and surgery have been tied together since the first operation was carried out. Throughout the history of surgery, the variety of materials used to close wounds has, included wires of gold, silver, iron, and steel; dried gut; silk; animal hairs; tree bark, and other plant fibers; and more recently, a wide selection of synthetic compositions [3].

Suture materials may be classified according to their behavior in tissue (absorbable or non-absorbable), structure (monofilament or multifilament), or their origin (synthetic, organic or metallic). Among suture materials, chromic gut and silk sutures are most commonly used for surgical wound closure in veterinary medicine [4]. Chromic gut is biologic, absorbable, multifilament suture [5]. It is treated and coated with chromium salts to increase its tensile strength, delay its absorption, and decrease its tissue reactivity [6]. It represents the standard with which modern materials are frequently compared [7]. Silk is a braided, naturally-occurring non-absorbable protein suture called fibroin fiber made by silkworm larva. The braided characteristic of silk allows for better handling, and it is defined as the gold standard suture material in terms of knot security. Silk leads to a significant reaction in tissues [6,8]. “Jimat” is a multifilament thread which is made of polyamide polymer nylon, manufactured to reinforce vehicle tyres [9].

All suture material implanted in a surgically created wound elicits varying degrees of tissue reactivity [10]. Implanted suture is recognized by the immune system as foreign material, and the inflammatory response is described as a foreign body reaction. The severity and duration of the reaction are affected by the type, amount, and longevity *in situ* of the suture, and the extent of tissue trauma produced by the surgical procedure. The inflammatory response is essential for normal wound healing to occur [11]. However, suture that elicits a severe and prolonged inflammatory reaction can hinder the healing process and make a wound more susceptible to infection. Therefore, the ideal suture would be one that remains only long enough for tissue tensile strength to be regained, whereas inciting an inflammatory response that is limited in duration and severity [12]. It should be non-electrolytic, non-capillary, non–allergenic, and non-carcinogenic [10].

Healing of muscle tissue after surgical repair is affected both by intrinsic and extrinsic factors that initiate an inflammatory response [13,14]. The intrinsic reaction is a measure of the natural immune response to injury while extrinsic factors include inflammatory response to the presence of a foreign body such as suture material at the site of repair. Therefore, tissue reaction to these materials is one of the crucial factors to be considered while choosing the best suture for the surgical repair [15].

Appropriate suture selection depends on an understanding of the physical and chemical properties of the suture, as well as suture-tissue interaction. Pliability refers to how easy the suture can bend. Multifilament sutures are braided or twisted and are more pliable, handling easier than monofilament sutures. The tissue reactivity to sutures is influenced by the physical and chemical characteristics of the material and the individual immune response [16].

Most of suture materials used for closure of wound edges are studied and well documented by many authors elsewhere in the world. Especially, numerous studies have been performed to assess the effects of suture materials on the skin during closure and wound approximation. As the field of veterinary surgery and diagnostic imaging is introduced and opened lately in Ethiopia, it is difficult to get documentations about sutures and suturing. Chromic gut and silk are sutures which are mostly used in Ethiopia to close wound edges of skeletal muscles and skin in large and small animals. But, due to the non-availability and expensiveness of such suture materials most of the veterinarians in Ethiopia used locally extracted, easily accessible, and cheapest suture material namely “jimat.” They use it to close most of the surgical wounds made on skeletal muscles and skin in many parts of the country. In Ethiopia, it is widely used as a suture material, but so far no studies have been undertaken about its reaction and acceptability by the tissue and the handling characteristics of “jimat” as a suture material at national level. It was hypothesized that, “jimat” suture material will produced an equal inflammatory reaction and suture handling characteristics with chromic gut and silk sutures in cat thigh muscle.

Therefore, the general objective of this study was:

- To evaluate the use of “jimat” as suture material in Veterinary Surgery.
- The specific objectives were:
- To evaluate the inflammatory response of skeletal muscle to chromic gut, silk, and “jimat” sutures.
- To evaluate the handling characteristic of chromic gut, silk, and “jimat” suture at the time of suturing.
- To compare and recommend a suture material which have least tissue reactivity among studied suture materials.

## Materials and Methods

### Ethical approval

For this particular experimental research, a total of 36 male cats were used. These animals were handled according to the guidelines of the Tigray National Regional State Science and Technology Agency Capacity Building, Research and Technology Development Directorate, Regional Research Ethics Committee. This study was conducted inside the laboratory and complete protocol of anesthesia was followed during surgical operations. As per the Regional Research Ethics Committee, the animals were fed and watered *ad libidum*. Also, the animals received chemoprophylactic drug injection to minimize post-operative complication. In addition to this, we followed all cats postoperatively until the animals were recovered well from artificially created surgical wound and discharged home uneventfully.

### Study area

This study was conducted from November, 2013 to April, 2014 in Kombolcha Animal Diseases Survey, Research and Diagnostic Laboratory located in Amhara regional state, South Wollo zone, 375 km far from Addis Ababa, Capital city, to the North East direction [17].

### Study animals

For experimental research many authors used different species of animals like mice [18], pigs [19], rabbits [20], dogs [21] or rats [22], respectively to assess the inflammatory reaction against various types of suture materials. In the current study, cats were used to evaluate tissue reactivity of different suture materials in *in vivo* study as Runk *et al*. [12] and Papazoglou *et al*. [23] did. For this particular study, a total of 36 healthy, intact male local breed cats were selected and randomly grouped as A, B, and C. Cats were handled according to the guidelines of the Tigray Regional Research Ethics Committee. Mean age of the cats was 10.02±1.81 month (range: 7.5-13 month) and mean weight was 1.8±0.36 kg (range: 1-2.5 kg). These animals were collected from voluntary owners. The owners of the cats were consented to participate in the study. All study animals were isolated and kept inside the laboratory in a separate cage one month before the commencement of the study. The cats were fed with similar diet (well inspected offals) and water *ad libidum* from a common source to standardize their nutrition and maintenance. While the study was over, health status of the cats was re-established, open castration was performed in all study animals, as an incentive, before they were dispatched back to the owners.

### Study design

This study was an experimental, randomized block design, where chromic gut was used as a comparative control and silk and “jimat” of identical size were used as test material.

### Suture material

“Jimat” thread was extracted from vehicle tyre. It was mainly used to repair shoe in Ethiopia. But, in order to compare “jimat” thread with other suture materials, it was very important to know from which materials it is made. Authors who were working on tyre fabric cord confirmed that nylon belt was made from either nylon or polyester [9]. There was no way to get information about the constituent of the “jimat” thread which is imported to Ethiopia. Therefore, we referred “jimat” thread to a textile engineering company to know the specific answer as to from which material “jimat” is made. We communicated with ADC Research and Development PLC, Addis Ababa, Ethiopia. Fiber identification of “jimat” was done by using burning and solubility tests in laboratory. After all tests were conducted, they confirmed that “jimat” thread, which is available anywhere in Ethiopia, is made from nylon.

**Figure-1:**
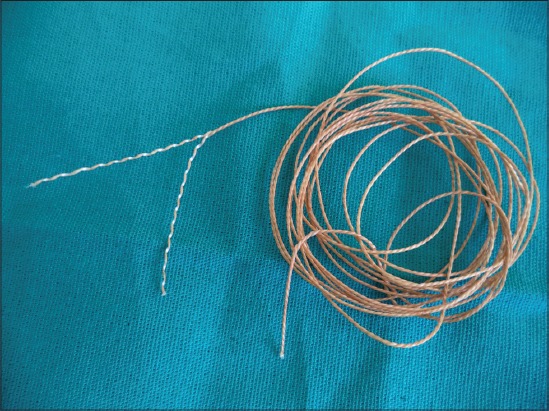
Picture of double strand “jimat” thread.

Ligations and closure of incised thigh muscle in cats were made only by chromic gut, silk, and “jimat” for Groups A, B, and C, respectively (Table-1). Silk (3-0) (Shandong Sinorgmed International Co., Ltd.) and chromic gut (3-0) (Hualyin Medical Instruments Co., Ltd.) were selected for suture implant, but for “jimat,” which is a double strand thread, had no recommended size as, it was manufactured for another purpose (Figure-1). So, for this particular study single strand of “jimat” thread were used and compared; since, it had almost similar thickness with 3-0 suture materials. Chromic gut and silk were threaded with a needle attached with the suture. But, “jimat” thread is not prepared with a needle. Therefore, to avoid this variation a reversed cutting eyed taper point type needle was used for all suture materials [24].

**Table-1:** Grouping of the animal sample.

Suture materials	Chromic gut	Silk	“Jimat”
Study groups	A	B	C

### Surgical procedure

Before the surgical procedures were carried out, experimental animals were withheld from food and water for 12 h. Premedication was achieved with atropine (0.02 mg/kg, IM), acepromazine (0.04 mg/kg, IM), and ketamine (2.0 mg/kg, IM) 20 min before induction. Induction and maintenance anesthesia were ketamine (10 mg/kg, IV) [12]. The cats were placed in left lateral recumbency and the lateral side of the right thigh was clipped, shaved and then washed with soap. The site of proposed incision was then washed with liquid savlon, disinfected with tincture iodine (2%) solution and draped with sterile towels.

**Figure-2:**
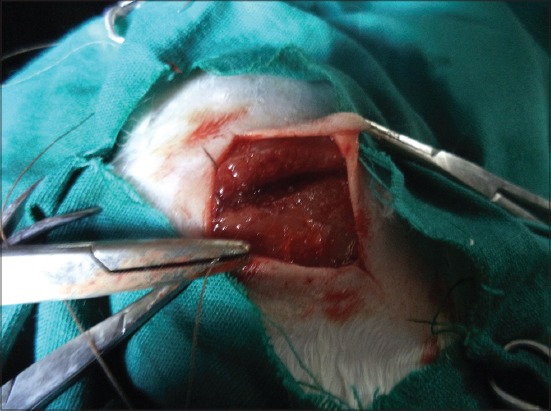
Surgical operation on cat thigh muscle: Vertical skin incision was made to expose biceps femoris muscle; surgical repair of exposed biceps femoris muscle with suture.

As Figure-2 illustrates, a vertical incision measuring 3 centimeters was made on the skin with a Bard-Parker scalpel blade (No. 15), followed by blunt dissection with metzenbaum scissors bilaterally to the incision line to expose the thigh muscle (biceps femoris). A 3 cm transverse incision was then made on the exposed muscle, perpendicular to the direction of the muscle fibers (Figure-2). The muscle was then repaired using the selected suture material depending on the group with simple interrupted suture pattern. Sterilized and packed suture materials were used for chromic gut and silk, but “jimat” thread was used after sterilizing in boiling water for 1 h. All sutures were placed 0.5 cm far from muscle edge and 1 cm each apart [25]. Sutures were tied using a Mayo-Hegar needle holder. With precise positioning, the sutures were tied with sufficient tension to loosely approximate wound edges and bring muscle into apposition. The skin closure was done with 3-0 monofilament nylon based on the consideration that nylon is relatively non-reactive and would not have a significant effect on the healing of the underlying muscle [26]. Double square knots were made in the usual manner. Care was taken to ensure that for all throws, tension on the ears were equal and opposite in direction. The ears, the cut ends of knot, were cut to a length of 4-5 mm. A single veterinary surgeon had performed surgical operations on right hind leg thigh muscle and suture materials were implanted to the incision. All operations were done by a single veterinary surgeon. Each animal received 20 mg/kg of intravenous cefazolin (Vifazolin; Fujisawa Pharmaceutical, Osaka, Japan) at the time of induction of anesthesia as a chemoprophylaxis [23]. Sterile saline water was applied daily as a local dressing [27]. On day 9, nylon sutures at incision site were removed in all three groups [23].

### Histological evaluation

At the end of the required time period, the cutaneous sutures were reopened. From all three groups’ a total of six cats, two from each, were randomly selected for day 1 sampling. The rest cats were allowed to stay in the group. At days 3, 7, 14, 21, and 28, two, two cats were randomly selected from each group until all cats sampled. The right thigh muscle containing the suture was excised by No. 11 blade for 3 cm length, 1 cm width, and 1 cm thickness and collected separately from each cat. After the required samples had collected, the remnant of excised muscle tissue was reapposed and each cat was transferred to another room where they were observed and treated daily.

The tissues immediately surrounding the sutured muscle along with the sutures were taken and preserved in 10% formalin solution. The representative samples were sectioned at four to 5 µ thickness, processed and stained with hematoxylin an eosin method [22] in Ayder referral hospital, pathology laboratory. Tissue reactions on response to the different suture materials at different time interval were studied histologically by a veterinary pathologist and compared. It (tissue reaction) could be clearly defined histologically, by evaluating the degree of leukocytic infiltration, and granulation and fibrous tissue formation (FTF) [28].

### Suture handling characteristics

During the suturing three handling characteristics were evaluated and noted in the protocol for each cat; the precision of knot tying (PKT), square knot positioning (SKP) and resistance to knot slippage (RKS). Each characteristic was rated by a surgeon, who was not involved in any of this surgical operation. Differences between the three groups of cats for each handling characteristic were statistically tested and p<0.05 was considered statistically significant [25].

### Data collection

Every cat which was involved in this study was given an individual unique number. All data concerning those cats were collected during and post-surgical periods.

### Histological samples

Leukocytic infiltration elicited by studied suture materials were estimated by counting the average number of 4 different types of cells (lymphocytes, neutrophils, macrophages, fibroblasts) in oil immersion field. The overall cellular density was assigned in a numerical grade from 0 to 3 using a modified Ehrlich-Hunt numerical scale (0 - Absence, 1 - Bare scattering, 2 - Moderate, 3 - Dense aggregation) [29,30]. All the tissue samples were evaluated by a single pathologist to avoid subjective variation in reporting. The evaluator had no previous knowledge as to which group of cats the slides represents. Each study group was given twelve numerical scores. A mean value was selected and standard deviation from the mean was calculated for each study-group. This gives a single numerical value that was used for inter-group comparisons. On top of that from three oil immersion fields of each sample one representative field was selected and the presence or absence of granulation and FTF was recorded separately (0 - Yes and 1 - No).

### Suture handling characteristics

Individual sample data on suture handling characteristics were recorded by rating the PKT, SKP, and RKS from 1 - poor to 5- excellent. Therefore, individual cats in each group had single numerical value for each characteristic. This value was used for further statistical analysis.

### Statistical analysis

The data were entered into Microsoft excel spreadsheet, coded appropriately and it was exported to Statistical Package for the Social Science (SPSS) accordingly. For analysis of the data, descriptive statistics using SPSS (version 17; SPSS Inc., Chicago, IL, USA) for Microsoft Windows (Microsoft Corporation, Redmond, Washington, USA) were used. The data were analyzed by using various tests. Two-way ANOVA was used to compare mean leukocytic infiltration among the studied group and at different days interval. For group wise comparison *post*-*hoc* test (Tukey test) were used. But for granulation and FTF, the percentage was analyzed and compared by using Chi-square test. Kruskal–Wallis test was used to compare the median values of handling characteristics of all suture materials. For all parameters, p<0.05 was considered as significant.

## Results

### Leukocytic infiltration

On macroscopic evaluation, the surgical sites in all cats looked healthy at 1, 3, 7, 14, 21, and 28 days postoperatively. Histological examination of tissue samples showed varying degree of leukocytic infiltration of three suture materials at different time (day) interval. Table-2 shows the least square mean of chromic gut, silk, and “jimat;” which indicates that Group A (4.5±1.9) elicited higher leukocytic infiltration than Group B (4.3±1.5) and Group C (2.4±1.2). This means chromic gut has higher tissue reactivity than silk and followed by “jimat.” The suture material showed a different degree of an inflammatory reaction at 6 different days interval (Table-2). All suture materials produced greater leukocytic infiltration on the 1^st^ day (5.2±1.5) than other days. On day 3 (5±1.7) greater inflammatory reaction was recorded than day seven. But, from day 14 onward on day 21 and 28 tissue reactivity against all suture materials was decreased as the time progressed and the mean values are 3.5±0.9, 2.3±1.2 and 1.7±0.6, respectively. The difference between least square mean of groups and days may or may not have significantly differed among suture groups and days.

**Table-2:** Least square mean (±SD) of chromic gut, silk, and “jimat” suture materials in groups and at different days of suture implantation on cat thigh muscle.

Variables	N	Mean±SD
Group		
A – Chromic gut	12	4.5±1.9
B – Silk	12	4.3±1.5
C – “Jimat”	12	2.4±1.2
Days		
1	6	5.2±1.5
3	6	5±1.7
7	6	4.8±1.2
14	6	3.5±0.9
21	6	2.3±1.2
28	6	1.7±0.6

N=Number of observation, SD=Standard deviation

As the ANOVA table indicates there was a significant difference in leukocytic infiltration among chromic gut, silk and “jimat” suture materials level (p<0.05) and suture implantation period (days) (p<0.05). Even the degree of leukocytic infiltration was different at different days interval of suture implantation period in cat muscle tissue (Table-3).

**Table-3:** The ANOVA result of leukocytic infiltration achieved from three different suture material groups and six different days in histopathological examination.

Source of variation	SS	Df	MS	F	p value
Day	33.46	5	6.692	28.68	0.000^b^
Group	16.33	2	8.17	35.00	0.000^c^
Residual	2.33	10	0.23		
Total	52.12	17			

b p value from comparison between 6 days of suture inoculation in two-way ANOVA.

c p value from comparison between three groups of suture in two-way ANOVA

Multiple *post*-*hoc* comparison among six different days interval indicated that there was no significant leukocytic infiltration difference between days 1, 3, and 7 (p>0.05) and between days 21 and day 28 (p=0.122). On the other days, there was a greater significant difference between the first 7 days and days 14, 21 and 28 (p>0.05) (Table-4). This indicates that all suture materials evoked relatively similar degree of leukocytic infiltration from day 1 to day 7 and between day 21 and day 28. But, they produced a different degree of inflammatory reaction between the first 7 days of suture incubation and the last few days of this study (from day 14 to day 28). In addition to this, no difference in the degree of leukocytic infiltration was recorded between day 21 and day 28 in all suture materials. As Table-2 illustrates, the least square mean of inflammatory reaction of the 1^st^ day specimens (5.2±1.5) of all types of suture materials revealed a neutrophilic infiltration at the perisutural area. Just adjacent to the sutural zone, a dense aggregate of inflammatory cells were frequently present. Inflammatory cells, mostly neutrophils were mainly observed during the early stage, the proportion of macrophages present compared to other cell types increased as time proceeded; especially chromic gut had high number of neutrophil cells relative to other sutures.

**Table-4:** Analytical comparisons of results in leukocytic infiltration achieved from three different suture materials at six different days in histopathological examination by *post-hoc* tests.

(T) days	(B) days	Mean difference (T-B)	SE	p value^a^
1	3	0.33	0.39	0.418*
	7	0.17	0.39	0.682*
	14	1.67	0.39	0.002**
	21	2.83	0.39	0.000**
	28	5.50	0.39	0.000**
3	7	−0.17	0.39	0.682*
	14	1.33	0.39	0.007**
	21	2.50	0.39	0.000**
	28	3.17	0.39	0.000**
7	14	1.50	0.39	0.003**
	21	2.67	0.39	0.000**
	28	3.33	0.39	0.000**
14	21	1.17	0.39	0.014**
	28	1.83	0.39	0.001**
21	28	0.67	0.39	0.122*

a Tukey test,

** Significant,

* Insignificant,

SE=Standard error

**Figure-3:**
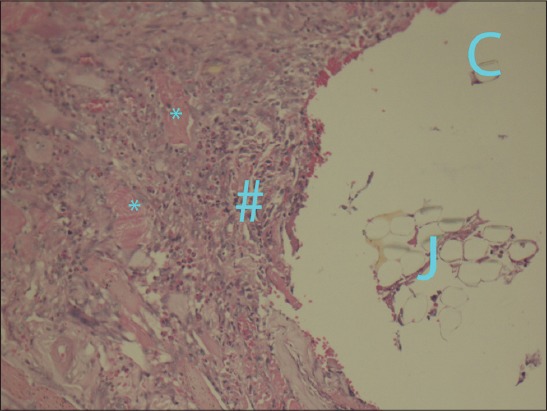
Dispersed inflammatory cells (#) in the supporting tissues of (a) silk suture material (F) (×20), (b) chromic gut suture (T) (×20), and (c) “jimat” suture material (J) (*granulation tissue) (×20) at day 7 specimens.

On 3^rd^ (5±1.7) and 7^th^ (4.8±1.2) day, it was observed that all sutures remained in place and similar type of inflammation was observed in the tissues having a mixture of macrophages and lymphocytes. In this study, the inflammatory reaction to suture materials was reached at its top on the 1^st^ and 3^rd^ day. Silk caused an inflammatory response that increased between 3 and 7 days and was the suture with the highest inflammation score at 7 days (Figure-3). Relatively higher macrophage infiltration was recorded at day 7 in all sutures.

**Figure-4:**
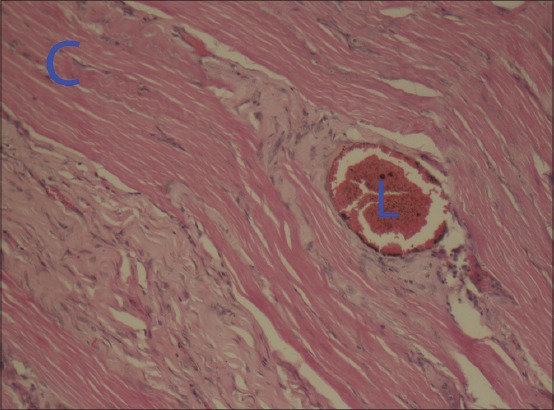
Photomicrograph at day 28 post-surgical showing minimal inflammatory reaction around sutures; (a) “Jimat” suture material showed healing and formation of fibrosis around the suture (×20), (b) Inflammatory reaction to silk suture (×20), (c) Chromic gut suture (×5) (L - Lumen, S - Silk Suture).

On 14 day (3.5±0.9), the inflammatory response to silk and chromic gut was relatively greater than “jimat” suture. But all suture materials elicited lower leukocytic infiltration, while it was compared with that of the previous days. Still chronic inflammatory cells, lymphocytes and macrophages and fibroblasts, were infiltrated around all suture materials. All tissue samples at day 21 (2.3±1.2) and day 28 (1.7±0.6) had minimal or no inflammatory component that could have been assessed to evaluate the healing process (Figure-4). Besides this, some macrophages and fibroblasts were identified around the suture lumen. The “jimat” suture had produced a mild to moderate, sustained inflammatory response over the study period. Moreover, there was no difference in the inflammatory reaction between the tissues surrounding the suture as time proceeded.

**Figure-5:**
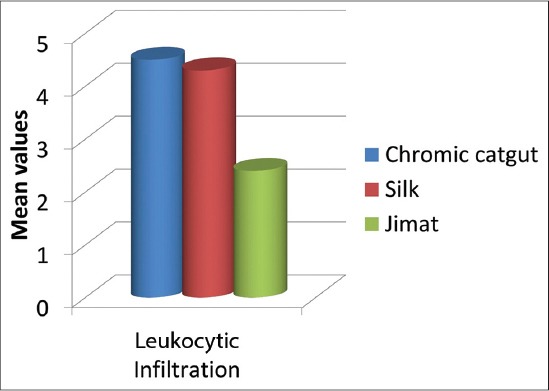
Comparison of mean values of inflammatory reaction elicited by chromic gut, silk, and “jimat” sutures in cat thigh muscle.

Multiple *post*-*hoc* suture material comparisons were performed by Tukey test to look differences among groups. The tests showed a significant difference between Groups A and C (p<0.05) and Groups B and C in both tests (p<0.05). But no significant difference was recorded between Groups B and C in Tukey test (p>0.05) (Table-5). This indicates that, there is a significant difference in leukocytic infiltration between chromic gut and “jimat” sutures and also there is a difference in tissue reactivity elicited by both silk and “jimat.” Contrary to this, there was no difference in leukocytic infiltration between chromic gut and silk in this experimental study. In order to identify which suture materials has less tissue reactivity; we should look to their least square mean. As Table-2 and Figure-5 illustrate, microscopic evaluation obtained from biopsy specimens: Group A (4.5±1.9) showed severe inflammation followed by Group B (4.3±1.5) and Group C (2.4±1.2) in descending order of severity of inflammatory response. Group C with “jimat” sutures showed the least response (Figure-4). So, chromic gut had elicited greater leukocytic infiltration followed by silk and “jimat.” Therefore, the overall results in leukocytic infiltration indicate that “jimat” had less tissue reactivity than silk and chromic gut (Table-2). The collagen fibers were seen at day 3 which were loosen, but it got organized while the time proceeded around day 14.

**Table-5:** Analytical comparisons of results in leukocytic infiltration achieved from three different suture materials in histopathological examination.

(I) Group	(J) Group	Mean difference (I-J)	SE	p value^c^
A	B	0.333	0.279	0.260
	C	2.167	0.279	0.000
B	C	1.833	0.279	0.000

c Tukey test.

SE=Standard error

### Granulation tissue formation (GTF)

**Figure-6:**
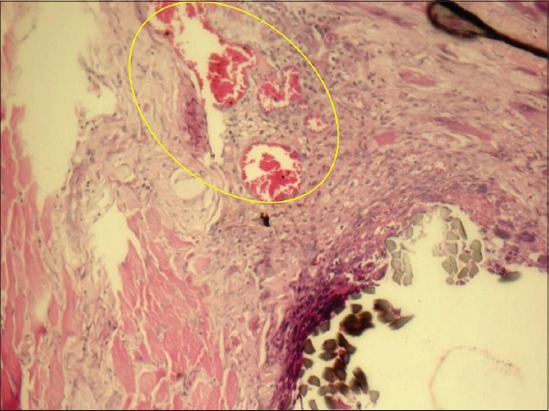
Granulation tissue formation (encircled) in silk suture at day 7 (×20).

Out of 36 tissue samples examined under three different groups, 22 (61.1%) had developed GTF. Out of 12 cats in Group A, which were sutured with chromic gut, 4 (33.3%) did not develop GTF; but in Groups B and C, 6 (50%) and 8 (66.7%) cats developed GTF around suture materials, respectively (Figure-6). The overall GTF was insignificant among groups of suture materials (p>0.05) (Table-6).

**Table-6:** The Chi-square value of granulation tissue formation in different suture materials in cat thigh muscle.

Predictor variable	Granulation tissue formation (N [%])		p value^a^

	No	Yes	
Group			
A	4 (33.3)	8 (66.7)	0.513
B	4 (33.3)	8 (66.7)	
C	6 (50)	6 (50)	
Overall	14 (38.9)	22 (61.1)	
Day			
1	6 (100)	0	0.027
3	0	6 (100)	
7	0	6 (100)	
14	2 (33.3)	4 (66.7)	
21	4 (66.7)	2 (33.3)	
28	6 (100)	0	
Overall	18 (50)	18 (50)	

a Chi-square test

Out of 36 cats which were examined for the presence of GTF at six different periods (days) of time, half of them i.e. 18 (50%) cats developed granulation and the remaining 18 didn’t develop GTF. GTF was fully developed in 6 (100%) cats, at 3^rd^ and 7^th^ day post-surgery (Figure-6). Also, at day 14, 4 (66.7%) cats showed granulation. On the other hand at first, 21 and 28 days after suture implantation, there was no GTF in 6 (100%), 4 (66.7%) and 6 (100%) cats, respectively. The overall GTF was significant between days of suture materials implantation (p=0.027) (Table-6).

### FTF

Out of 36 cats examined in all groups examined, 14 (38.9%) had developed fibrous tissue and 22 (61.1%) of them did not develop fibrous tissue. At group level, cats in Group A, which were sutured with chromic gut, and Group B showed no development of fibrous tissue 10 (83.3%) (Figure-5); whereas, 2 (16.7%) of them had developed marked FTF around the suture materials. On the other side majority of cats, i.e., 10 (83.3%), in Group C, sutured with “jimat,” showed marked FTF around the suture material (Figure-5). The overall FTF was significantly different among groups (p=0.024) (Table-7).

**Table-7:** The Chi-square value of fibrous tissue formation in different suture materials in cat thigh muscle.

Predictor variable	Fibrous tissue formation (N [%])		p value^a^

	No	Yes	
Group			
A	10 (83.3)	2 (16.7)	0.024
B	10 (83.3)	2 (16.7)	
C	2 (16.7)	10 (83.3)	
Overall	22 (61.1)	14 (38.9)	
Day			
1	6 (100)	0	0.553
3	4 (66.7)	2 (33.3)	
7	4 (66.7)	2 (33.3)	
14	4 (66.7)	2 (33.3)	
21	4 (66.7)	2 (33.3)	
28	2 (33.3)	4 (66.7)	
Overall	22 (61.1)	14 (38.9)	

a Chi-square test

Out of the 36 cats examined for the presence of FTF at six different days of interval after suture implantation, 22 (61.1%) did not develop fibrosis; whereas, 14 (38.9%) propagated FTF. The overall FTF was insignificant among days of examination (p>0.05). At the day level interval there was a marked FTF on 28 days with a similar score of 4 (66.7%) after suturing (Figure-5). But, on the other days including first (6 [100%]) and on 3^rd^, 7^th^, 14 and 21 days there was no FTF, and values were equal (4 [66.7%]) (Table-7).

### Suture handling characteristics

Table-8 shows the median value of suture handling characteristics of Groups A, B, and C. All suture groups were tested with Kruskal–Wallis test for PKT, SKP, and RKS. But as the individual and overall median indicates there is no difference in suture handling characteristics among all suture materials (p>0.05).

**Table-8:** Comparison of median value of suture handling characteristics of chromic gut, silk, and “jimat” sutures.

Variables	Suture material	N	Median±SD	p value^a^
PKT	A	12	5±0.81	0.345
	B	12	5±0.79	
	C	12	5±1.07	
	Total	36	4.53±0.81	
SKP	A	12	4.5±0.49	0.393
	B	12	5±0.78	
	C	12	5±0.56	
	Total	36	4.56±0.83	
RKS	A	12	5±0.00	0.335
	B	12	5±3.89	
	C	12	5±3.89	
	Total	36	5±3.89	
Overall		36	4.64±0.47	0.347

a Kruskal–Wallis test,

N=Number of observation, SD=Standard deviation, PKT=Precision of knot tying, SKP=Square knot positioning, RKS=Resistance to knot slippage

## Discussion

A number of studies have been performed to assess the effects of suture materials on the skin during closure and wound approximation, while little literature is available on the response of skeletal muscle to the sutures. Healing of muscle tissue is different from other tissues, because of the presence of satellite cells which are myogenic precursor cells, located between the basal lamina and the plasma membrane of individual myofibers. They proliferate and differentiate into multinucleated myotubes and eventually into myofibers themselves. Interactions between inflammatory cells and skeletal muscle cells can influence muscle cell proliferation, differentiation and injury [13,14].

The healing of a wound is a complex biological process controlled by several variables. When sutures are inserted, the reaction is essentially local. These involves two components; one being the reaction to the trauma inflicted by the suture needle, and the other reaction to the material used for suturing. The former is, as expected, a universal reaction of non-specific nature and of short duration. The reaction to suturing material, on the other hand, is variable, being dependent on the nature of the suture material. This usually persists for a prolonged period till either the sutures are removed or are absorbed [31,32].

Tissue reaction to suture materials is a crucial factor in choosing the best suture material. A thorough understanding of the physical, mechanical, and chemical properties of the commonly used suture materials is vital to the clinical practice of surgery [33].

### Leukocytic infiltration

Inflammatory reaction caused by the suture materials is one of the crucial factors that alter the healing process. It has been suggested in literature that an indirect method of assessing the healing process in a tissue sample is by studying the extent of inflammatory reaction during healing [34]. The density of different inflammatory cells especially neutrophils, lymphocytes, macrophages and fibroblasts are the best indicators of the degree and extent of inflammation caused by suture materials [22].

In the present study, the acute neutrophilic reaction was seen on the 1^st^ day. The infiltration of leukocytes was higher in all sutures. This finding is similar with Selvig *et al*. [35] and Özçaka *et al*. [36]. This higher leukocytic infiltration on the 1^st^ day might be due to suture trauma when a needle and sutures are passed through the tissue [35].

In the current study, from day three to seven the leukocytic infiltration was higher. In addition to this, the predominant cells were lymphocytes and mainly macrophages. The nature of the local reaction showed considerable variation not only to different suture materials but also to the same material in different animals. This indicates that the tissue reaction tended to be directed more to the suture material. In general, the initial acute neutrophilic reaction commenced declining and was replaced by a chronic reaction. This was evidenced by a reduction in the number of neutrophils and their replacement by lymphocytes and macrophages. The degree of leukocytic infiltration was gradually reduced as the time proceeded. Silk showed higher leukocytic infiltration at the 7^th^ day. “Jimat” elicited relatively minimal tissue reactivity than others throughout the study period. Similar results were obtained by several authors, who studied the tissue reactivity to plain gut, chromic gut, silk, nylon and PDS suture materials [14,30,35,36].

At day 7, in this study, higher macrophage infiltration was recorded. The presence of macrophages in healing muscle enhances the repair process by creating a conducive atmosphere for the optimal healing of skeletal muscle [14,30,37]. Persistence of high macrophage content from 3 to 7 days post-surgical with gut in this study might be due to the scavenging of gut collagen fibers which has beneficial effect on muscle healing [22].

In the case of silk, it caused higher leukocytic infiltration at day 7. This result is analogous with previous workers in fish [38] and in the dog [39]. It could be due to a factor that the body response to residual sericin, waxes or silicones used in the manufacture of the sutures is higher at day 7 post-implantation [40].

In our study, all suture materials did not elicit significantly different leukocytic infiltration at day 1, 3, and 7 which is analogous with Özçaka *et al*. [36]. The least square means in the current study indicated that there was a difference between the first 7 days and the rest of study period in leukocytic infiltration. All sutures except “jimat” elicited higher infiltration of cells which were decreased markedly as the time proceeded especially started from day 14. This finding is similar with Setzen and Williams [41]; Wainstein *et al*. [21] and Kim *et al*. [39].

In the present study, overall leukocytic infiltration in Group A with chromic gut sutures was severe, followed by the Group B with silk suture. Group C with “jimat” sutures showed minimal inflammatory response relative to other study groups. There was also a significant difference in leukocytic infiltration between “jimat” and chromic gut and between “jimat” and silk. Therefore, “jimat” stimulated minimal tissue reactivity than chromic gut and silk. Similar results were obtained by several authors with different type of suture materials including chromic gut, silk, and nylon (“jimat”) [34,38,39,42,43].

As cited above, in this study, chromic gut provoked sever inflammatory reaction than “jimat” and relatively similar with silk suture. This finding corroborates with many authors who studied tissue reactivity of chromic gut in different species of animals and tissues implanted [4,21,34,38,44].

In this study, the response of the skeletal muscle to the silk material resembles with most of the observations made on animals or humans, in which silk has been considered to be a material inducing unwanted tissue reactions [42,45-48]. Many studies have reported that silk sutures are more susceptible to bacterial invasion and severe tissue inflammatory reactions compared to other suturing products [35,49].

In addition to its chemical properties the braided configuration of the silk sutures (by wicking effect) encouraged microbial contamination of the whole surface of wound just 3 days after suturing. This is may be the reason why silk suture caused significant tissue reactions in many species of animals, while it was compared with other suture materials like “jimat” [50,51].

In the present study, “jimat” had elicited the least inflammatory reaction than both chromic gut and silk sutures. Researchers, who assessed the tissue reactivity of nylon, confirmed that it had elicited the least tissue reactivity than other sutures compared, such as chromic gut, silk, and polypropylene [5,39,47].

While it was compared with chromic gut, in this study, it stimulated the least tissue reactivity. Their mean value was 4.5 for chromic gut and 2.4 for “jimat.” As their mean indicates there was a great difference on ability to elicit inflammatory reactions. This difference was highly significant (p<0.05). Chromic gut had elicited almost double of the tissue reaction elicited by “jimat.” Even if, the type of leukocytes infiltrated around the suture were similar; there was a great difference in a number of leukocytes elicited by both sutures throughout the study period. As it is discussed above, chromic gut was known by its greater inflammatory reaction than sutures it compared with [34,47,52,53].

While “jimat” is compared with silk, still it has elicited slightest tissue reaction. In the present study, silk had a mean value of 4.3 which is almost similar with chromic gut and much greater than “jimat” (2.4). This difference was highly significant (p<0.05). Our finding showed that the tissue reaction stimulated by silk was much higher than the inflammatory reaction elicited by “jimat.” As it was briefly discussed before, silk was recognized by its ability to produce high tissue reactivity while it was implanted in animals and humans tissue [45-47,54].

In addition to this, earlier studies showed that nylon (“jimat”) suture had caused relatively more inflammatory response in muscle tissue or internal organs than external (skin) [22,55]. In our study, “jimat” stimulated least tissue reactivity relatively than chromic gut and silk in cat thigh muscle. Therefore, as Ribeiro *et al*. [55] and Bhargava *et al*. [22] indicated, it is possible to conclude that, “jimat” will elicit less inflammatory reaction in skin than it will elicited in muscle. So, as it stimulated least tissue reactivity in both muscle tissue and skin, it is promising to recommend for wound approximation on skin and muscle tissue. This finding was also supported by Kim *et al*. [39] who identified higher inflammatory reaction on mucosal layers than in keratinized tissues.

Prior to this study, silk was the “inexpensive” suture material as compared to other non-absorbable suture materials [56]. In terms of cost-effectiveness, “jimat” is the most cheap suture material than other sutures included in this study. Therefore, in addition to its least tissue reactivity, it is also choosy suture materials in its cost-effectivity and easy accessibility.

### Granulation and FTF

Angiogenesis is critical to wound repair. Newly formed blood vessels participate in provisional GTF and providing nutrition and oxygen. In addition, new vessels are involved in the delivery of inflammatory cells that transmigrate through the endothelial basement membrane to enter the site of injury. An abundant blood supply during wound healing helps to meet the enormous local demands of debridement, fibroblast proliferation, extracellular matrix synthesis, and epithelialization [57].

In the present study, all suture materials showed development of GTF (all scores >50%). Silk and chromic gut showed more GTF than “jimat.” But, the difference was insignificant (p>0.05). This result is ­analogous with Bellenger [58], who did with nylon. This might be due to the disturbances in the transmission of nervous pulses [59], pain [32] and lithogenesis [60]. In addition to this chromic gut treated and coated with chromium salts used to promote the proliferation of granulation tissue in wound healing [58].

In this experimental study, GTF was highly observed from day 3 to day 14 in all suture materials. This is characterized by proliferation of fibroblasts and newly formed blood vessels. The collagen fibers were loosened at the day 3 but it got organized around day 14. This finding in the present experiment corroborates with findings of Andrade *et al*. [32]; Coker [61] and Bernis-Filho *et al*. [62].

Our findings showed that there is a significant difference among suture materials in FTF. From all sutures examined “jimat” had developed high FTF (83.3%), whereas, chromic gut and silk showed least FTF. But, there was no significant difference among sutures at different days interval (p>0.05). Even if there is no difference in FTF at different day’s interval, all sutures showed higher FTF at day 28 (66.7%). This result is similar with previously published works of Runk *et al*. [12] and Ribeiro *et al*. [55].

In the present study, silk and chromic gut showed least FTF. This might be a justification for why they caused the delayed healing [48]. It is also an indicator of the presence of unresolved and consistent inflammatory reaction [12].

For non-absorbable sutures tissue reaction in internal organs will be ended with encapsulation of the suture material by fibrous tissue [35]. But, the foreign body reaction, consisting mainly of macrophages and or foreign body giant cells, may persist at the tissue implant interface for the lifetime of the implant [63].

### Suture handling characteristics

It is clear that the objective of suturing is to place multiple layers of tissues in close contact so that a minimal quantity of new connective tissue will be required to restore structural integrity of the tissue in the shortest possible time. Best sutures should have to have good physical characteristics beside their least inflammatory reaction elicited against [64,65]. Therefore, the physical characteristics of surgical sutures are one of the most important considerations in suture selection [66].

To characterize the physical and handling properties of suture materials, earlier published works showed use of highly sophisticated machines namely servohydraulic mechanical testing machine (858 Bionix Test) System [67]; Bionix 809 Axial/Torsional Test System with Test Star II digital controller [68]; which are not available in Ethiopia. Therefore, the researcher had decided to evaluate the handling characteristics of sutures by grading the PKT, SKP and RKS from 1 (poor) to 5 (excellent). Matičić *et al*. [25] conducted a comparative study of skin closure in dogs with polypropylene and polyglactin 910 and they recorded the average grade score of physical handling characteristics of each suture. They tried to compare the grade scores of sutures with each other. But, their interpretation was more personal than statistical and even they didn’t reach to conclusion. In such cases, if there are no prior studies, it is possible to take the average value as a cutting point, in which all sutures handling characteristics were compared with. Therefore, for this particular study, median ± standard deviation value of chromic gut, silk, and “jimat” sutures were preferred to compare with the average number of the grade score, cutting point, i.e. 3. Beside this the interpretation will be for those sutures scoring <3 are sutures with poor handling performance and for those sutures scoring >3 will be interpreted as sutures with excellent handling characteristics as per the finding of Schisterman *et al*. [69].

In this study, the median score for all sutures, chromic gut, silk, and “jimat,” was 5 which is above the cutting point 3. In addition to this, the p value showed that there is no significant difference between chromic gut, silk, and “jimat.” This means all sutures have equal performance with regard to PKT. Even the total median of these three sutures was 4.53, which was much higher than the cutting point. This indicated that chromic gut, silk, and “jimat” had very good PKT and there was no individual difference between sutures with their PKT performance. This result is supported with previously published work of Szarmach *et al*. [70].

Excellent results were found for all sutures in SKP with values of 4.5, 5, and 5 for chromic gut, silk, and “jimat,” respectively. Even if there was a slight difference between chromic gut and silk and “jimat;” all scores were above the cutting point and the difference was insignificant. Even the total median value of all sutures was 4.56, which was highly greater than the average point. Therefore, the performance of chromic gut, silk, and “jimat” sutures in SKP is excellent and similar with McFadden [71], who concluded that multifilament sutures like silk and nylon has improved handling characteristics and knotting ability and Charbit *et al*. [72] who showed silk can be taken as gold standard suture material in terms of knotting and knot security. The median score of chromic gut, 4.5, was relatively lower than silk and “jimat;” this might be due to the fact that natural multifilament twisted sutures, such as chromic gut, tend to act more like monofilaments than braided multifilament sutures [73] in which monofilament sutures have poor handling characteristics than multifilament sutures [71].

Knot strength is calculated by determining the force necessary to cause a knot to slip [74,75]. The least reliable part of any suture is the knot. In this study, a median value, 5, was scored for chromic gut, silk, and “jimat” sutures in RKS. All sutures scored similar value and even the total median score was 5. These scores are much better and higher than the cutting value. Therefore, it is possible to say that the performance of all sutures is equal and excellent with regard to RKS. This finding is corroborates with Charbit *et al*. [72] who evaluated silk suture material by using the “pull-out friction test” and McFadden [71] in multifilament nylon.

The present study also indicated that the overall median values for PKT, SKP and RKS for all suture materials had no significant difference and their score was 4.64. This is much higher than the average cutting value i.e. 3. Thus indicates that chromic gut, silk and “jimat,” sutures had similar performance in handling characteristics. In addition to this, the overall median score indicated that all sutures had much higher performance in all suture handling characteristics parameters. These findings are in agreement with published works of Fossum [8]; Roush [76] and McFadden [71].

## Conclusion

The present study revealed that a single strand “jimat” thread was elicited least tissue reactivity than both silk and chronic gut, which are known by their unwanted inflammatory reaction on animal tissue. Moreover, “jimat” suture had showed higher FTF than others. This may be an indicator of resolving of suture induced inflammatory reaction and being replaced by histologically normal fibrous connective tissue. The present study also showed that “jimat” had excellent suture handling characteristics as similar with the gold standard suture material, silk. In addition to this, it is easily availability throughout the country with the cheapest cost. Thus, in this particular study, “jimat” appears to be the most satisfactory suture material as regards to both tissue reaction and suture handling characteristics. Its side effects were not studied as a biomaterial; therefore, further studies should be conducted to know about the carcinogenic effect of “jimat” thread.

## Authors’ Contributions

TB, AT and APB designed the study. TB: Collection of samples, execution of the experimental study, collation and analysis of data, interpretation of the results, and drafting the manuscript. AT: Read the microscopic slides. APB and AT provided technical guidance and participated in scientific investigations and discussion. All authors read and approved the final manuscript.
